# Cognitive impairment in Chinese patients with cervical dystonia

**DOI:** 10.3389/fneur.2022.961563

**Published:** 2022-09-16

**Authors:** Kuncheng Liu, Yanbing Hou, Ruwei Ou, Tianmi Yang, Jing Yang, Wei Song, Bi Zhao, Huifang Shang

**Affiliations:** Department of Neurology, West China Hospital, Sichuan University, Chengdu, China

**Keywords:** cervical dystonia, cognitive impairment, visuospatial function, education, motor symptoms

## Abstract

**Objective:**

Cognitive impairment (CI) in patients with cervical dystonia (CD) has been reported in many studies but with inconsistent findings. We investigated the prevalence, characteristics, and clinical factors related to CI in Chinese patients with CD.

**Methods:**

Sixty-eight patients with CD and 68 healthy controls (HCs) were included in the study. Demographic and clinical data were investigated. A logistic regression analysis was conducted to discriminate the clinical factors associated with CI in patients with CD. A cluster analysis was performed to explore the different characteristics within the group of CD patients with CI.

**Results:**

We found that 42 (61.76%) patients with CD had CI. The most frequent CI domain was visuospatial function (39.71%), followed by memory (38.24%), attention/working memory (29.41%), language (25.00%), and executive function (23.53%). CD patients with CI were older, less educated, had an older age of onset, more severe motor symptoms and disability, and experienced more pain than CD patients without CI. The presence of CI in patients with CD was associated with less education (OR = 0.802, *p* = 0.034) and a higher Toronto Western Spasmodic Torticollis Rating Scale (TWSTRS) severity subscore (OR = 1.305, *p* = 0.001). The cluster analysis identified two different subgroups of patients, one with relatively mild cognitive impairment and the other with relatively severe cognitive impairment.

**Conclusion:**

CI is relatively common in Chinese patients with CD, with the most common CI domain of the visuospatial function. In the present study, CI in patients with CD was associated with less education and more severe motor symptoms, and patients with CI may be further divided into two subgroups based on different extent and domain of cognitive decline.

## Introduction

Dystonia is a neurological condition presenting with sustained or intermittent muscle contractions that can cause abnormal involuntary movements (1). According to the distribution of affected body parts, dystonia can be classified into focal, segmental, multifocal, generalized, and hemidystonia (1). Cervical dystonia (CD) is the most common type of focal dystonia, with a prevalence of 4.98/100,000 (2), and is characterized by involuntary contraction of the neck muscles, resulting in abnormal movements and postures of the head, neck, and shoulders (3).

Currently, the pathogenesis of CD remains unclear, and treatment options are limited. Apart from motor symptoms, non-motor symptoms such as depression, anxiety, cognitive impairment (CI), and sleep disorders, also affect the quality of life of patients with CD (4). CI was first reported in 1994 among patients with CD and manifested as impaired visuospatial function (5). A number of subsequent studies found that patients with CD have impairments across multiple cognitive domains, such as prospective memory (6), language function (7), and executive function (8), indicating that CI may be associated with the pathogenesis of CD within the central nervous system. However, some studies did not find any difference between patients with CD and healthy controls (HCs) with respect to cognitive function (9–11). Moreover, there are inconsistent findings on the correlative factors of CI in patients with CD. Some studies found that CI was associated with non-motor symptoms such as depression and anxiety (12, 13), while other studies found that CI was independent of motor and other non-motor symptoms of CD (14, 15). Therefore, we planned to perform a multi-domain, cross-sectional study to investigate the prevalence, characteristics, and clinical factors related to CI in Chinese patients with CD.

## Patients and methods

### Patients

In this study, 68 (23 male and 45 female) idiopathic patients with CD who were diagnosed according to the published criteria (1) and 68 (23 male and 45 female) HCs were recruited between April 2020 and April 2022 from the Department of Neurology, West China Hospital, Sichuan University. This study adhered to the principles of the Declaration of Helsinki. The ethics committee of West China Hospital of Sichuan University approved the study (No. 2022-260). All participants provided written informed consent before their inclusion in the study. All 68 patients had focal CD, and none of them had segmental, multifocal, or generalized dystonia. Patients diagnosed with acquired dystonia according to medical and drug histories, neurological examination, brain magnetic resonance imaging (MRI), and laboratory tests were excluded from the study. For genetic analysis, whole exome sequencing (WES) was performed in 44 of the total 68 (64.71%) patients with CD; no pathogenic, likely pathogenic, or risk genes associated with dystonia were identified. The remaining 24 (35.29%) patients declined WES because of economic or privacy reasons. None of the patients had other concomitant neurological disorders or positive family history of neurological conditions such as Parkinson's disease (PD), Alzheimer's disease (AD), or other forms of dementia. If patients had a history of botulinum toxin injection; were on oral medication such as benzhexol and/or clonazepam, or had undergone any procedure that may have affected the cognitive function within the last 6 months, they were excluded from the study. Patients who had <6 years of education were also excluded. The HCs were matched using the stratification method based on the sex, age, and educational level of the patients with CD. None of the HCs had a history of neurological disorders or positive family history of neurological disorders.

### Clinical and cognitive assessments

Demographic and clinical features, including sex, age, age of onset, disease duration, education level, and treatment regimen were collected by professional neurologists in a face-to-face interview.

The dystonic symptom of CD was assessed by the Toronto Western Spasmodic Torticollis Rating Scale (TWSTRS), which was divided into three sections: severity, disability, and pain (16). Depression was assessed by the Hamilton Depression Scale (HAMD; 24 items) (17) and the Beck Depression Inventory (BDI) (18). Anxiety was assessed by the Hamilton Anxiety Scale (HAMA) (19). Excessive daytime sleepiness was assessed by the Epworth Sleepiness Scale (ESS) (20). Fatigue was assessed by the Fatigue Severity Scale (FSS) (21).

Global cognitive function was assessed by the Chinese version of Addenbrooke's Cognitive Examination Revised (ACE-R) (22). Thus far, there are no universal diagnostic criteria for cognitive disorders in CD or other forms of dystonia. Most previous studies on CI in patients with CD have simply used the published cut-off scores of global cognition tests such as the Mini-Mental Status Examination (MMSE) or the Montreal Cognitive Assessment (MoCA) (9, 10), or performed a direct comparison (Student's *t*-test) of specific cognition test scores between patients and controls (6, 7, 11, 23). In the current study, to get a comprehensive as well as a precise picture of CI in patients with CD, we referred to the criteria for other neurological disorders like PD-mild cognitive impairment (PD-MCI) (24). Furthermore, to evaluate the specific function in different cognitive domains, we conducted a series of neuropsychological assessments including 10 tests representing five cognitive domains: (i) memory (Hopkins Verbal Learning Test-Revised [HVLT-R] and Brief Visuospatial Memory Test-Revised [BVMT-R]); (ii) attention/working memory (Adaptive Digit Ordering Test [DOT-A] and Backward Digit Span Test [DST]); (iii) language (Wechsler Intelligence Scale for Adult-Chinese Revised [WAIS-RC] and Boston Naming Test [BNT]); (iv) executive function (Verbal Fluency Test [VFT] and Clock Drawing Test [CDT]); and (v) visuospatial function (Benton Line Orientation [BLO] and Clock Copying Test [CCT]) (25). Finally, in this study, we defined CI in patients with CD as a total score < (HCs' mean −1.5 standard deviations [SD] of the HCs') in (i) at least two tests in one domain or (ii) one test per domain in at least two domains based on the criteria for PD-MCI (24). The ACE-R total score was used as a global reference of cognitive status and not as part of the CI definition given its lack of sensitivity and specificity.

### Statistical analyses

All analyses were conducted on SPSS 23.0 and MatLab R2021b. GraphPad Prism 9 was used to create the figures. All tests were two-tailed, and a *p-*value < 0.05 was considered to indicate statistically significant differences. Continuous data were presented as mean ± SD. Categorical data (sex) were presented as exact numbers. Demographics, clinical characteristics, and cognitive assessments of patients and HCs as well as CD patients with and without CI were compared using Student's *t*-test for continuous data and a chi-squared test for categorical data (sex). Then, a multivariate binary logistic regression was used to determine the clinical factors associated with CI in patients with CD. The presence or absence of CI was used as the dependent variable. Age, the age of onset, education, and the three subdivisions (severity, disability, and pain) of TWSTRS were used as covariates, which were selected based on the significant results (selection criteria: *p-*value < 0.05) from comparisons between CD patients with and without CI. After the regression analysis, variables with a *p*-value < 0.05 were considered as the potential risk factors of CI in patients with CD. The Hosmer–Lemeshow test was used to validate the regression model, and a *p*-value > 0.05 was considered to have reliable goodness of fit. The Spearman's correlation test was used to analyze the potential correlation between the covariates, and a *p*-value > 0.05 was considered to indicate no correlation between the two variables.

We performed a non-hierarchical (k-means) cluster analysis to explore the different patterns of CI within the group of CD patients with CI on the basis of multi-domain cognitive tests. In case of overweighting a single feature in the clustering solution, we used composite indicators, each of which comprised two cognitive tests concerning the same domain. Therefore, we included the following: (i) an attention/working memory domain; (ii) an executive function domain; (iii) a language domain; (iv) a memory domain; and (v) a visuospatial function domain. The averaged *Z*-score of each domain was used for cluster analysis. To locate the optimal number of clusters, we calculated the Calinski–Harabasz pseudo-*F*-value for different cluster models, respectively, for which a higher pseudo-*F*-value was indicative of a better cluster model. During the data-driven clustering process, the *F*-value represents the different contributions of variables, and a higher *F*-value indicated a more distinct difference in the variables between clusters. Analysis of variance (ANOVA) and Student's *t*-test were used for continuous data, and the chi-squared test was used for categorical data (sex) in a *post-hoc* analysis of the subgroups generated by cluster analysis. To clarify the different features of CI in different subgroups of patients, we compared the patients' absolute scores on 10 assessment scales in five specific cognitive domains with normative cut-off scores generated by HCs during the *post-hoc* analysis.

## Results

**Figure 1:**
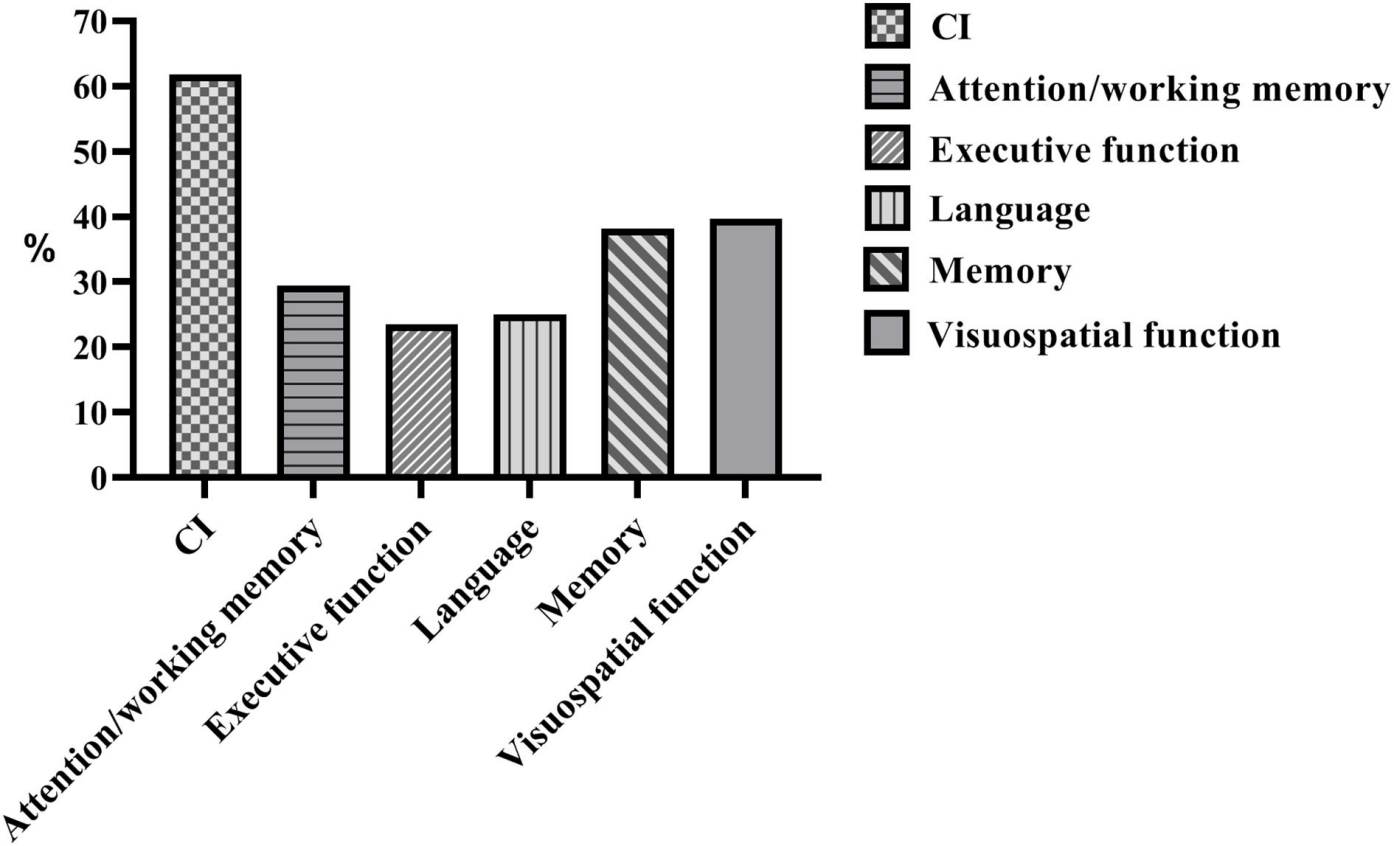
The frequencies of abnormal performances in global and individual cognitive domains of patients with CD. CI, cognitive impairment.

Normative data of HCs on cognitive assessments are listed in Table 1. After comparing the 10 specific cognitive assessment scores between patients with CD and matched HCs, we found that 42 (61.76%) CD patients presented with CI. The most frequent CI domain was visuospatial function (39.71%), followed by memory (38.24%), attention/working memory (29.41%), language (25.00%), and executive function (23.53%) (Figure 1). Among the 42 CD patients with CI, only one (2%) patient had a single domain impairment (visuospatial function), while the remaining 41 (98%) patients had multiple domain impairments.

**Table 1:** Normative data of HCs on cognitive assessments.

	**Mean ±SD**	**Range**	**Cut-off score**
Sex (M/F)	23/45	-	-
Age (years)	45.44 ± 12.78	22–75	-
Education (years)	12.13 ± 2.92	6–19	-
ACE-R (Max 100)	90.13 ± 6.33	80–99	80/81
**Attention/working memory**			
DOT-A (Max 12)	7.34 ± 1.69	4–12	4/5
DST (Max 18)	8.19 ± 2.10	4–13	5/6
**Executive function**			
VFT (Max -)	19.38 ± 3.99	12–27	13/14
CDT (Max 15)	13.68 ± 1.53	10–15	11/12
**Language**			
WAIS-RC (Max 26)	19.06 ± 3.21	13–26	14/15
BNT (Max 30)	25.78 ± 2.68	20–30	21/22
**Memory**			
HVLT-R (Max 36)	26.26 ± 3.97	18–36	20/21
BVMT-R (Max 36)	28.38 ± 4.03	19–36	22/23
**Visuospatial function**			
BLO (Max 30)	27.77 ± 2.29	22–30	24/25
CCT (Max 15)	14.62 ± 0.69	13–15	13/14

HC, healthy control; M, male; F, female; SD, standard deviations; ACE-R, Addenbrooke's Cognitive Examination Revised; DOT-A, adaptive digit ordering test; DST, backward digit span test; VFT, verbal fluency test; CDT, clock drawing test; WAIS-RC, Wechsler intelligence scale for adult-Chinese revised; BNT, Boston naming test; HVLT-R, Hopkins verbal learning test-revised; BVMT-R, brief visuospatial memory test-revised; BLO, Benton line orientation; CCT, clock copying test.

The comparisons between patients with CD and HCs are listed in Table 2. Patients and HCs were identical with respect to the number of participants, sex distribution, age, and educational level. However, patients with CD showed poor performance in all 11 cognitive assessment scales, including global cognition and the five different cognitive domains.

**Table 2:** Comparison between patients and HCs.

	**HCs**	**Patients**	***p-*value**
Number of participants	68	68	-
Sex (M/F)	23/45	23/45	1
Age (years)	45.44 ± 12.78	45.22 ± 12.61	0.919
Education (years)	12.13 ± 2.92	11.78 ± 3.26	0.508
ACE-R	90.13 ± 6.33	82.00 ± 12.21	<0.001^*^
**Attention/working memory**			
DOT-A	7.34 ± 1.69	5.43 ± 2.31	<0.001^*^
DST	8.19 ± 2.10	6.74 ± 3.02	0.001^*^
**Executive function**			
VFT	19.38 ± 3.99	15.50 ± 3.82	<0.001^*^
CDT	13.68 ± 1.53	10.91 ± 2.34	<0.001^*^
**Language**			
WAIS-RC	19.06 ± 3.21	14.76 ± 4.31	<0.001^*^
BNT	25.78 ± 2.68	23.10 ± 4.26	<0.001^*^
**Memory**			
HVLT-R	26.26 ± 3.97	20.00 ± 5.65	<0.001^*^
BVMT-R	28.38 ± 4.03	17.44 ± 9.05	<0.001^*^
**Visuospatial function**			
BLO	27.77 ± 2.29	23.70 ± 3.61	<0.001^*^
CCT	14.62 ± 0.69	12.85 ± 1.99	<0.001^*^

HC, healthy control; M, male; F, female; ACE-R, Addenbrooke's Cognitive Examination Revised; DOT-A, adaptive digit ordering test; DST, backward digit span test; VFT, verbal fluency test; CDT, clock drawing test; WAIS-RC, Wechsler intelligence scale for adult-Chinese revised; BNT, Boston Naming Test; HVLT-R, Hopkins verbal learning test-revised; BVMT-R, brief visuospatial memory test-revised; BLO, Benton line orientation; CCT, clock copying test.

* Significant difference.

The comparisons between CD patients with and without CI are listed in Table 3. CD patients with CI were older, less educated, older at the time of disease onset, had more severe motor symptoms and disability, and experienced more pain than CD patients without CI. However, there were no significant intergroup differences with respect to sex distribution, disease duration, sleepiness, fatigue, depression, and anxiety.

**Table 3:** Demographic and clinical data between CD patients with and without CI.

	**Total**	**With CI**	**Without CI**	***p*-value**
Number of patients	68	42	26	-
Sex (M/F)	23/45	13/29	10/16	0.525
Age (years)	45.22 ± 12.61	48.45 ± 12.62	40.00 ± 10.91	0.006^*^
Age of onset (years)	40.76 ± 12.30	43.79 ± 12.16	35.87 ± 11.08	0.009^*^
Education (years)	11.78 ± 3.26	10.62 ± 3.21	13.65 ± 2.40	<0.001^*^
Disease duration (years)	4.46 ± 4.41	4.66 ± 4.48	4.13 ± 4.35	0.633
TWSTRS	32.10 ± 12.58	37.51 ± 11.79	23.36 ± 8.23	<0.001^*^
Severity	17.09 ± 4.89	19.29 ± 4.37	13.54 ± 3.41	<0.001^*^
Disability	11.32 ± 6.42	13.52 ± 6.47	7.77 ± 4.54	<0.001^*^
Pain	3.68 ± 3.69	4.70 ± 3.84	2.05 ± 2.80	0.003^*^
ESS	3.25 ± 3.85	3.29 ± 3.55	3.19 ± 4.37	0.923
FSS	24.03 ± 15.77	23.14 ± 16.03	25.46 ± 15.54	0.560
HAMA	6.90 ± 4.63	6.69 ± 4.51	7.23 ± 4.90	0.644
HAMD	7.82 ± 5.72	7.90 ± 5.94	7.69 ± 5.45	0.883
BDI	5.41 ± 4.43	5.17 ± 4.64	5.81 ± 4.13	0.566

CD, cervical dystonia; CI, cognitive impairment; M, male; F, female; TWSTRS, the Toronto Western Spasmodic Torticollis Rating Scale; ESS, Epworth Sleepiness Scale; FSS, Fatigue Severity Scale; HAMD, Hamilton Depression Scale; HAMA, Hamilton Anxiety Scale; BDI, Beck Depression Inventory.

* Significant difference.

Comparisons of cognitive assessment results between CD patients with and without CI are presented in Table 4. CD patients with CI showed poor performance in all 11 cognitive assessment scales, including global cognition and the five different cognitive domains.

**Table 4:** Cognitive assessments between CD patients with and without CI.

	**Total**	**With CI**	**Without CI**	**p-value**
ACE-R	82.00 ± 12.21	75.62 ± 11.05	92.31 ± 4.51	<0.001^*^
**Attention/working memory**				
DOT-A	5.43 ± 2.31	4.60 ± 2.19	6.77 ± 1.86	<0.001^*^
DST	6.74 ± 3.02	5.21 ± 2.21	9.19 ± 2.48	<0.001^*^
**Executive function**				
VFT	15.50 ± 3.82	14.05 ± 3.34	17.85 ± 3.40	<0.001^*^
CDT	10.91 ± 2.34	9.95 ± 2.42	12.46 ± 1.03	<0.001^*^
**Language**				
WAIS-RC	14.76 ± 4.31	12.83 ± 4.08	17.88 ± 2.45	<0.001^*^
BNT	23.10 ± 4.26	20.90 ± 3.61	26.65 ± 2.43	<0.001^*^
**Memory**				
HVLT-R	20.00 ± 5.65	17.62 ± 5.49	23.85 ± 3.38	<0.001^*^
BVMT-R	17.44 ± 9.05	13.64 ± 8.60	23.58 ± 5.91	<0.001^*^
**Visuospatial function**				
BLO	23.70 ± 3.61	21.77 ± 3.07	26.81 ± 1.79	<0.001^*^
CCT	12.85 ± 1.99	11.86 ± 1.91	14.46 ± 0.51	<0.001^*^

CD, cervical dystonia; CI, cognitive impairment; ACE-R, Addenbrooke's Cognitive Examination Revised; DOT-A, adaptive digit ordering test; DST, backward digit span test; VFT, verbal fluency test; CDT, clock drawing test; WAIS-RC, Wechsler intelligence scale for adult-Chinese revised; BNT, Boston naming test; HVLT-R, Hopkins verbal learning test-revised; BVMT-R, brief visuospatial memory test-revised; BLO, Benton line orientation; CCT, clock copying test.

* Significant difference.

As for the potential risk factors of CD patients developing CI, the multivariate logistic regression model indicated that less education (OR = 0.802, 95%CI: 0.640–0.904, *p* = 0.034) and a higher TWSTRS severity subscore (OR = 1.305, 95%CI: 1.112–1.532, *p* = 0.001) were associated with the presence of CI. Regarding the statistical diagnostics, the *p*-value of the Hosmer–Lemeshow test for this regression model was 0.786, which showed reliable goodness of fit. The *p-*value of the Spearman's correlation analysis for the variable “educational level” and the “TWSTRS severity subscore” was 0.473, which showed no correlation between the two variables.

The results of cluster analysis within the group of CD patients with CI are listed in Table 5. During the cluster analysis, we tested two-, three-, and four-cluster models, respectively, and the two-cluster model had the highest Calinski–Harabasz pseudo-*F*-value (two-cluster model, 14.70; three-cluster model, 14.13; four-cluster model, 13.76), representing the best cluster solution. In the two-cluster solution, Cluster I included 23 patients with CD and Cluster II included 19 patients with CD. Demographic and clinical data between the two clusters are presented in Table 6. The sex distribution, disease duration, pain, sleepiness, fatigue, depression, and anxiety were not different between clusters. However, patients in Cluster II were older, less educated, older at the time of disease onset, and had more severe motor symptoms and motor disability than patients in Cluster I. Comparisons of cognitive assessments between the two clusters are presented in Table 7. Among the 11 cognitive assessments, only three (VFT, CDT, and CCT) were not different between the two clusters. Patients in Cluster II had poorer performance in the remaining eight cognitive assessments than those in Cluster I.

**Table 5:** Association of clusters with variables included in the cluster analysis.

	**Cluster I (RMCI)**	**Cluster II (RSCI)**		
	**Z score**		**Z score**			
	**Mean**	**SD**	**Mean**	**SD**	**F**	***p*** **-values**
Attention/working memory	0.49	0.90	−0.59	0.54	20.84	<0.001^*^
Executive function	0.10	0.60	−0.12	0.71	1.16	0.288
Language	0.56	0.62	−0.67	0.61	41.37	<0.001^*^
Memory	0.48	0.64	−0.58	0.66	27.89	<0.001^*^
Visuospatial function	0.31	0.60	−0.38	0.94	8.35	0.006^*^

RMCI, relatively mild cognitive impairment; RSCI, relatively severe cognitive impairment; SD, standard deviations.

* Significant difference.

**Table 6:** Demographic and clinical data between two clusters.

	**Cluster I (RMCI)**	**Cluster II (RSCI)**	***p*-value**
Number of patients	23	19	-
Sex (M/F)	8/15	5/14	0.555
Age (years)	41.65 ± 10.30	56.68 ± 10.11	<0.001^*^
Age of onset (years)	38.10 ± 10.58	50.68 ± 10.42	<0.001^*^
Education (years)	11.87 ± 2.96	9.11 ± 2.88	0.004^*^
Disease duration (years)	3.55 ± 3.73	6.00 ± 5.03	0.078
TWSTRS	32.75 ± 12.05	43.26 ± 8.67	0.003^*^
Severity	17.65 ± 4.55	21.26 ± 3.26	0.006^*^
Disability	11.30 ± 6.94	16.21 ± 4.73	0.012^*^
Pain	3.79 ± 3.47	5.79 ± 4.06	0.094
ESS	3.91 ± 4.37	2.53 ± 2.06	0.186
FSS	20.35 ± 14.47	26.53 ± 17.52	0.218
HAMA	6.57 ± 5.03	6.84 ± 3.92	0.846
HAMD	8.00 ± 6.32	7.79 ± 5.61	0.910
BDI	5.39 ± 5.18	4.89 ± 4.01	0.728

RMCI, relatively mild cognitive impairment; RSCI, relatively severe cognitive impairment; M, male; F, female; TWSTRS, the Toronto Western Spasmodic Torticollis Rating Scale; ESS, Epworth Sleepiness Scale; FSS, Fatigue Severity Scale; HAMD, Hamilton Depression Scale; HAMA, Hamilton Anxiety Scale; BDI, Beck Depression Inventory. ACE-R, Addenbrooke's Cognitive Examination Revised.

* Significant difference.

**Table 7:** Cognitive assessments between two clusters.

	**Cluster I (RMCI)**	**Cluster II (RSCI)**	***p*-value**
ACE-R	82.70 ± 7.47	69.47 ± 11.71	0.001^*^
**Attention/working memory**			
DOT-A	5.70 ± 2.01	3.26 ± 1.59	<0.001^*^
DST	6.26 ± 2.26	3.95 ± 1.35	<0.001^*^
**Executive function**			
VFT	14.62 ± 3.63	13.84 ± 3.04	0.722
CDT	10.30 ± 2.60	9.53 ± 2.17	0.305
**Language**			
WAIS-RC	15.00 ± 3.50	10.21 ± 3.10	<0.001^*^
BNT	23.00 ± 2.73	15.37 ± 2.87	<0.001^*^
**Memory**			
HVLT-R	20.91 ± 3.22	13.63 ± 5.02	<0.001^*^
BVMT-R	18.74 ± 8.36	9.89 ± 7.49	0.009^*^
**Visuospatial function**			
BLO	22.91 ± 2.53	20.39 ± 3.16	0.007^*^
CCT	12.35 ± 1.67	11.26 ± 2.05	0.066

RMCI, relatively mild cognitive impairment; RSCI, relatively severe cognitive impairment; ACE-R, Addenbrooke's Cognitive Examination Revised; DOT-A, adaptive digit ordering test; DST, backward digit span test; VFT, verbal fluency test; CDT, clock drawing test; WAIS-RC, Wechsler intelligence scale for adult-Chinese revised; BNT, Boston naming test; HVLT-R, Hopkins verbal learning test-revised; BVMT-R, brief visuospatial memory test-revised; BLO, Benton line orientation; CCT, clock copying test.

* Significant difference.

As for the two clusters in the current study, the *F*-value represents the different contributions of variables during the clustering process, wherein the higher the *F*-value, the more distinct the differences in the variables between the two clusters (Table 5). Specifically, the *F*-values were relatively low for the visuospatial and executive function domains, indicating the relatively small difference of these two domains between the two clusters. Furthermore, the absolute scores of the visuospatial and executive function assessment scales (Table 7) showed a relatively mild decline (>75% cut-off scores by HCs) in both clusters. However, the much higher *F*-value in attention/working memory, language, and memory domain indicated the more distinct difference in these domains between the two clusters. After comparing the absolute scores of assessment scales in these three domains (Table 7), we found that patients in Cluster I had relatively mild impairments (>75% cut-off scores by HCs), while those in Cluster II had relatively severe impairments ( ≤ 75% cut-off scores by HCs) in the attention/working memory, language, and memory domains. As mentioned before, there are yet no universal criteria for cognitive disorder in CD or other forms of dystonia. Therefore, we defined patients in Cluster I as those with CD with a relatively mild CI subtype (CD-RMCI), because the impairments in all five domains were relatively mild when compared with the HCs (>75% cut-off scores by HCs). By contrast, patients in Cluster II had relatively severe impairments in three domains ( ≤ 75% cut-off scores by HCs) with only two domains having relatively mild impairments (>75% cut-off scores by HCs); hence, we defined patients in Cluster II as having CD with a relatively severe CI subtype (CD-RSCI).

## Discussion

To our knowledge, this study has a relatively large sample size and includes some comprehensive multi-domain cognitive assessments to investigate the prevalence and clinical characteristics of CI in Chinese patients with CD. The prevalence of CI in these patients (61.76%) is relatively high, and the most frequent CI domain was visuospatial function (39.71%). We found that CI was associated with less education and more severe motor symptoms in CD, and patients with CI could be divided into CD-RMCI and CD-RSCI by cluster analysis.

Based on previous reports, the proportion of patients with CD who presented with CI ranged from 0 to 56.14% (10, 15). Such discrepancies may attribute to the following fact: no CI reported in some studies may be due to using simple global cognition tests like MMSE or MoCA (9, 10); varied different frequencies of CI reported in some studies may be because only a single cognitive domain was explored, such as frequencies of visuospatial dysfunction (23), executive dysfunction (26), and memory disorder (6). In our research, we performed a series of detailed cognitive assessments including multiple cognitive domains, which may be more specific than only using global cognition tests, and more comprehensive than only concentrating on one single domain. We found that the most frequent cognitive impairment domain was visuospatial function, which was in line with a number of studies using different assessing methods (5, 23, 27–29). In the meantime, deficits in other domains like memory (6), attention/working memory (14), language (7), and executive function (26) were also found in previous reports.

Studies have proven that basal ganglia are involved in visuospatial processing, and movement disorders like CD that have major basal ganglia dysfunction may produce distinct patterns of visuospatial impairment (30). Recent studies have proposed that the pathophysiology of CD may exceed beyond the basal ganglia, and structures like the cerebellum may also be a part of the network disruption during the pathogenesis of dystonic symptoms in CD (31–33). A recent brain network localization study on CD found network lesions scattered throughout the cerebellum, brainstem, and basal ganglia (34). The abnormal connectivity between the cerebellum and somatosensory regions may encompass the lesion locations causing dystonic symptoms in patients with CD (34). Besides motor symptoms, the complex network of the cerebellum and other regions may also be involved in cognitive dysfunction in CD (35). As the cerebellum influences the integration of received visual feedback signals when processing future actions, it may also be partly why over a third of patients with CD experience visuospatial impairments (35). In the future, functional MRI studies targeting the visual-cognition network are needed to explore the underlying mechanism of visuospatial impairments in CD.

Memory disorder was not common in previous studies on CD. As mentioned above, no impairment in the memory domain was reported in previous studies applying simple global cognition assessments (10, 36). In the current study, we used HVLT-R and BVMT-R to test both verbal memory and non-verbal memory and found a number of CD patients with memory disorders. According to previous studies on hyperkinetic movement disorders, the impairment in the memory domain in patients with CD was possibly related to basal ganglia dysfunction and might involve the frontal/prefrontal-basal ganglia pathways (6, 37). Similar to motor control, the role of basal ganglia in memory was to participate in the selection and extraction procedure of memory data stored in other regions (38, 39). The basal ganglia dysfunction in CD may cause problems in the aforementioned procedure, leading to memory disorders (6). This issue deserves further investigation in future functional neuroimaging studies in patients with CD.

Several studies have reported attentional impairment in generalized dystonia and focal dystonia (40, 41). Similarly, patients with CD were reported to have altered functional activity in the dorso-lateral-prefrontal loop, which may be involved in attention impairment (42, 43). Moreover, several studies have reported language function impairment in CD and other types of focal dystonia like blepharospasm (44–46), which may indicate a connectivity dysfunction between the basal ganglia and the precuneus, thalamus, and frontal areas (44).

Executive dysfunction was common in previous studies on idiopathic dystonia. In the current study, nearly 25% of CD patients presented with executive dysfunction. The cortical-basal ganglia circuits were reportedly not only involved in the pathophysiology in CD but also linked to the domain of the executive function (7). Additionally, previous studies have demonstrated that the frontal areas are mainly responsible for the initiation and execution of an action plan, while the cerebellum might be critical for the regulation during the executing process; both structures are understood to be crucial to the normal executive function (11, 35, 40, 47). The executive dysfunction might be the consequence of altered neural activity in the cerebellum and frontal cortex in addition to the basal ganglia in CD (34).

In our study, CI in patients with CD was associated with less education, which was in agreement with numerous studies on cognition (48). Low education level has been one of the most widely accepted risk factors for CI and dementia (48). A meta-analysis has quantitatively evaluated the association between education level and risk of dementia, which reported that the dementia risk was reduced by 7% for a per year increase in education, showing a trend of a dose-response relationship between education and risk of dementia (49). Furthermore, the three subgroups of patients with CD (CD without CI, CD-RMCI, and CD-RSCI) exhibited the same pattern of the dose–response relationship between educational level and the extent of CI. Specifically, CD patients without CI had the highest level of education (13.65 ± 2.40 years), followed by CD-RMCI (11.87 ± 2.96 years), while CD patients with RSCI had the lowest level of education (9.11 ± 2.88 years).

In the present study, we found that CI in patients with CD was associated with a great TWSTRS severity subscore, which suggests that CI may occur in CD patients with more severe motor symptoms. Spearman's correlation analysis showed no correlation between the TWSTRS severity subscore and education level, which means they were independent factors, and the association between motor symptoms and CI was not affected by education level. As mentioned above, the association between CI and other motor and non-motor symptoms in CD was rather controversial (12–15). Some authors proposed that CI in patients with CD was unrelated to motor symptoms, and CI may precede the onset of dystonic symptoms, indicating an independent role of CI in the pathophysiology of CD (8, 14, 46). Others argued that CD may be a complex syndrome affecting multiple brain regions which are responsible for CI and other non-motor symptoms rather than a “pure” motor disorder (10, 12, 15). A recent meta-analysis failed to conclude the association between CI and other factors in CD owing to the small sample size and high heterogeneity of results (45). Yet, in our study, CI in patients with CD was highly associated with more severe motor symptoms, and this association was still valid between CD-RMCI and CD-RSCI in the subgroup analysis. Changes in the striatal-frontal circuits may be involved in both dystonic movements of the muscle and visuospatial impairments in CD (50). Besides, several studies have reported that the cerebellum and cerebello-basal ganglia interaction in patients with dystonia seemed to not only involve motor symptoms but also cognition (36, 51, 52). To conclude, the association between motor severity and CI indicates that there may be more widespread alterations beyond the basal ganglia, possibly including the frontal/prefrontal cortex and the cerebellum, which contribute to the pathophysiology of both motor symptoms and CI in patients with CD (42). The motor and non-motor regions may interact with each other during the development of the disease, resulting in different extents of motor impairment and CI (45). Future studies are needed to clarify the underlying mechanisms of the association between motor severity and CI.

As for the cluster analysis on CD patients with CI, both clusters had relatively mild impairments in the visuospatial and executive function domains. However, the extent of impairments in the attention/working memory, language, and memory domains were distinctly different between the two clusters, which was also the main reason why CD patients with CI could be divided into two distinct subgroups in this study. Although the motor severity and disability were different between clusters, the similar extent of the decline in the visuospatial and executive function in both clusters indicates that impairments in the brain regions and circuits associated with visuospatial and executive function may be independent of the development of motor symptoms of CD to a certain extent. Combined with the results of previous studies that CI may precede the onset of dystonic symptoms (8, 14), there is a possibility that the impairment in visuospatial and executive function may play an independent role in the pathophysiology of CD (46), while more broad cortical impairments causing decline in other cognitive domains may also participate in the motor development of CD (42). Whether patients in the CD-RMCI subgroup will have cognitive deterioration in memory, attention, and language domain and further develop into RSCI as the disease progresses remains unclear and requires longitudinal investigations.

It is a conventional view that CD is usually a non-degenerative neurological disorder. Although the disease duration was not significantly different between the two clusters (*p* = 0.078) in the present study, the CD patients with RSCI had a longer average disease duration (6.00 years) than those with RMCI (3.55 years). A recent study reported a negative correlation between gamma-aminobutyric acid-A (GABA_A_) receptor availability in the thalamus of patients with CD and disease duration, suggesting that a decompensating process of neurotransmission might be involved in the central mechanism of CD and may contribute to disease progression over time (53). A study on deep brain stimulation (DBS) found that long disease duration in patients with CD may interfere with the therapeutic effect of globus pallidus internus (GPi) stimulation (54). Combined with our study that CD patients with RSCI had longer disease duration than those with RMCI, it is possible that the severity of CI in patients with CD may aggravate with disease duration, and RSCI might represent a marker of disease progression. This hypothesis would counter the commonly accepted view that CD is not a progressive disorder. However, a recent meta-analysis failed to detect the association between CI and disease duration in CD because of the small sample size and high heterogeneity of results (45). Therefore, longitudinal observations of cognition status in CD patients with large sample sizes are required in future prospective studies.

In addition, although age or age of onset was not associated with CI in patients with CD in the current study, it is a fact that patients with RSCI were the oldest, followed by patients with RMCI, and patients without CI were the youngest, which suggests that aging may also be involved in the development of CI in patients with CD. A previous systematic review reported that CI was more common in the elderly population and the risk of CI increases with age (55). However, all of the studies on CI in patients with CD, including the current study, did not find an association between age and CI (6, 7, 12–15, 23, 26, 28, 29, 36, 56), indicating that the underlying pathophysiology of CI in patients with CD may be more complex than the common age-related cognition decline. Moreover, we used strictly matched HCs by the stratification method based on sex, age, and educational level, and the patients still showed worse cognitive performance than HCs, which suggests that cognitive disturbances might be more influenced by disease than age. It is known that age-related cognitive decline is already evident in middle age (>45 years old) and the incidence of CI increases with age (55, 57); among patients in the CD-RSCI group, in the present study, 18 patients (95%) were over 45 years old and only one patient (5%) was under 45 years old. Conversely, among patients in the CD-RMCI group, only seven (30%) were over 45 years old, while 16 (70%) were under 45 years old. It is possible that the CI in patients with CD may comprise both age-related pathophysiology and dystonia-related pathophysiology, and age-related CI pathophysiology may play a more important role in older patients than in younger patients. Therefore, further longitudinal studies with long-term follow-ups for motor symptoms and cognitive performance combined with neuroimaging approaches will be helpful to clarify the relationship between age-related cognitive decline and dystonia pathophysiology-related cognition decline in CD, and further differentiate the pattern between the two CI processes.

To summarize, the multi-domain impairments in cognition and the positive association between motor severity and CI indicate that the pathophysiology of CI in patients with CD was more of a network disorder than merely a result of some single region alterations. Specifically, the basal ganglia dysfunction and abnormal connectivity within the cortical-basal ganglia circuits, together with other functional changes like the cerebellum and their interconnected cortical and subcortical structures, may contribute to the development of CI in patients with CD. Motor symptoms may interact with CI, and education/aging may play a protective/aggravating role during the pathological process, respectively.

Our study has some limitations. First, this study was based on a cross-sectional design, which means that the findings need to be further validated by longitudinal studies. Second, the normative data from the 68 HCs were limited because of the sample size, and it would be better if the comparison was performed with the data from a larger healthy cohort. Third, results from the cluster analysis may be affected by the choice and number of variables included as well as the number of clusters sought. However, we used composite indicators in case of overweighting a single feature in the clustering solution, and if we include too many variables in the cluster analysis, the results may be confounded, because of the relatively small number of patients included (42 CD patients with CI). We also calculated the Calinski–Harabasz pseudo-*F*-value to locate the optimal number of clusters, which indicated the two-cluster model as the optimal solution for this study.

## Conclusion

CI is relatively common in Chinese patients with CD, with the most frequent CI domain of the visuospatial function. In the present study, CI in patients with CD was associated with less education and more severe motor symptoms, and patients with CI may be further divided into two subgroups by different extent and domain of cognitive decline.

## Data availability statement

The raw data supporting the conclusions of this article will be made available by the authors, without undue reservation.

## Ethics statement

The studies involving human participants were reviewed and approved by the Ethics Committee of West China Hospital of Sichuan University (No. 2022-260). The patients/participants provided their written informed consent to participate in this study.

## Author contributions

KL designed the study and drafted the manuscript. YH, RO, TY, and JY did the investigation, screened patients, performed assessments, and collected the data. WS and BZ did the statistical analyses. HS reviewed and edited the manuscript. All authors contributed to manuscript revision, read, and approved the submitted version.

## Funding

This study was supported by the Sichuan Science and Technology Program (Grant No. 2022ZDZX0023).

## Conflict of interest

The authors declare that the research was conducted in the absence of any commercial or financial relationships that could be construed as a potential conflict of interest.

## Publisher's note

All claims expressed in this article are solely those of the authors and do not necessarily represent those of their affiliated organizations, or those of the publisher, the editors and the reviewers. Any product that may be evaluated in this article, or claim that may be made by its manufacturer, is not guaranteed or endorsed by the publisher.
